# Self-assembly of a short-chain ionic liquid within deep eutectic solvents†

**DOI:** 10.1039/c7ra13557b

**Published:** 2018-02-20

**Authors:** Manoj Kumar Banjare, Kamalakanta Behera, Manmohan L. Satnami, Siddharth Pandey, Kallol K. Ghosh

**Affiliations:** School of Studies in Chemistry, Pt. Ravishankar Shukla University Raipur 492 010 Chhattisgarh India kallolkghosh@yahoo.com +91-771-2262583 +91-771-2263146; Centre for Interdisciplinary Research in Basic Sciences, JMI Jamia Nagar New Delhi 110 025 India; Department of Chemistry, Indian Institute of Technology Delhi Hauz Khas New Delhi 110 016 India

## Abstract

Ionic liquids (ILs) and deep eutectic solvents (DESs) are receiving increased attention from both academic and industrial research due to their immense application potential. These designer solvents are environmentally friendly in nature with tunable physicochemical properties. In the present investigation, we have studied the aggregation behavior of a short-chain IL 1-butyl-3-methylimidazolium octylsulphate [Bmim][OS] within aqueous DESs using fluorescence, UV-vis, dynamic light scattering (DLS) and FT-IR spectroscopic techniques. We have prepared two DESs, ChCl–urea and ChCl–Gly, which are obtained by heating a mixture of an ammonium salt choline chloride with hydrogen bond donor urea or glycerol, respectively, in 1 : 2 molar ratios. The local microenvironment and size of the aggregates are obtained from steady state fluorescence (using pyrene and pyrene-1-carboxaldehyde as polarity probes) and DLS measurements, respectively. DLS results shows that IL [Bmim][OS] forms relatively larger micelles within the aqueous solution of DES ChCl–urea (avg. hydrodynamic radii = 209 nm) than compared to ChCl–Gly (avg. hydrodynamic radii = 135 nm). A significant decrease in the critical micelle concentration and increase in the aggregation number (*N*_agg_) are observed within DES solutions as compared to that in water, thus indicating that the micellization process of the IL [Bmim][OS] is much favored in the DES solutions. Molecular interactions of [Bmim][OS] in DESs are revealed from FT-IR spectroscopic investigation. Furthermore, these systems were applied to study the IL-drug binding of the antidepressant drug promazine hydrochloride (PH).

## Abstract 2

Self-assembly of short-chain imidazolium-based ILs within DESs have been investigated by fluorescence, UV-Vis, DLS and FT-IR spectroscopy. Further, these micellar systems [Bmim][OS]-DESs are utilized to study the IL-drug binding of an antidepressant drug (PH).

## Graphical abstract

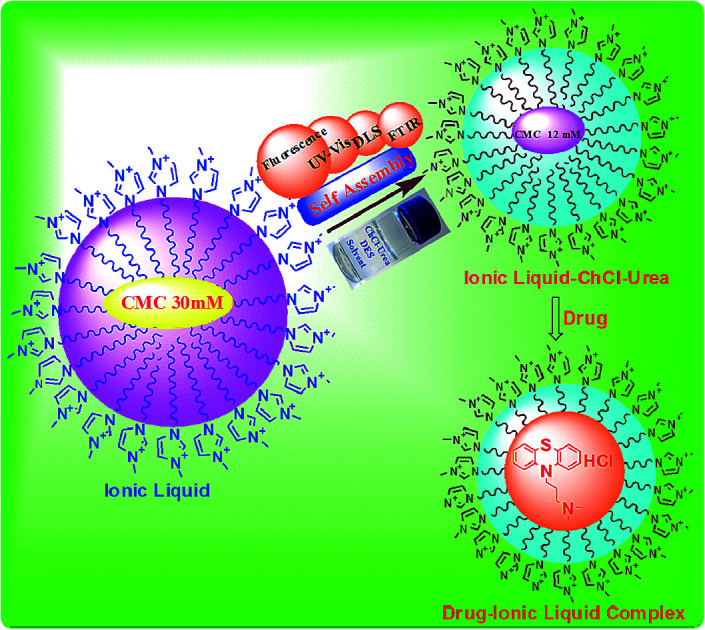

## 1. Introduction

Ionic liquids (ILs) possess unusual physicochemical properties and bright application potential in various fields.^1^ Surface active ILs as a novel class of surfactants are of significant interest to researchers worldwide and have stimulated more significance due to their self-assembling behavior.^2–5^ It has been reported that these ILs can display surface activity when dissolved in water, denoted by a decrease in the surface tension.^6–10^ It is noteworthy that the ILs have analogous properties to surface active agents and are capable of forming micellar nano-aggregates in aqueous solution.^11,12^ ILs show impressive physicochemical properties, such as, high thermal stability, high electrical conductivity, low vapor pressure and low melting points, *etc.*^13,14^ Furthermore, deep eutectic solvents (DESs) are emerging as new type of green solvents and analogs of ILs.^15^ Indeed, a DES generally comprises of two or three components that self-associate through hydrogen bonding to form a eutectic mixture and possesses melting point below that of the isolated components, low cost, less toxicity, high conductivity, relatively low viscosity, non-flammability, environmentally friendliness and biodegradability.^16–19^ The characteristics of DESs depend on its components, the ammonium salt and the hydrogen bond donors.^20–22^

The simple structure of short-chain IL based surfactants has produced a significant deviation in their micellar properties.^23^ It is important to have a clear picture on the micellization and interfacial behaviour of short-chain IL based surfactants to concern them effectively in particular fields.^23–25^ Several methods, such as, electrical conductivity, surface tension, dynamic light scattering (DLS), fluorescence, UV-visible and NMR spectroscopic techniques has been successfully utilized to study their micellization and interfacial behavior.^24–27^ Due to their structural flexibility and outstanding properties, short-chain IL based surfactant systems have generated immense significance, which is revealed in the increasing number of applications that have been reported in recent years.^25–27^ Aggregation behavior of surface active compounds including ILs within DESs have now become growing research interest, the number of publications as dedicated to the use of DESs for this purpose is rapidly increasing.^27–32^ Zhang *et al.*^30^ have investigated the aggregation behavior of 1-alkyl-3-methylimidazolium chloride with DESs (choline chloride and glycerol in a 1 : 2 molar ratio) by different techniques including fluorescence probe response, small angle X-ray scattering (SAXS) and FT-IR spectroscopy. They have presented a clear picture on the critical micellar concentration, micellar size and intermolecular interactions in IL/DES solutions using various spectroscopic techniques. Further, Pandey *et al.*^31^ have studied the self-aggregation of an anionic surfactant sodium dodecyl sulphate (SDS) within DESs. They have used surface tension, DLS and SAXS, density and dynamic viscosity measurements, fluorescence probe behavior of pyrene and 1,3-bis(1-pyrenyl)propane to characterize these molecular aggregates. Jackson *et al.*^22^ have investigated the aggregation of alkyltrimethylammonium bromides in choline chloride: glycerol DES by means of surface tension, X-ray and neutron reflectivity and small angle neutron scattering. Arnold *et al.*^32^ have investigated the self-assembly of anionic surfactant sodium dodecyl sulfate (SDS) within DESs, choline chloride/urea using X-ray reflectivity (XRR), small angle neutron scattering (SANS) and interestingly, the results propose that the micelle formation in DES solutions does not have the similar shape and size as those observed in water.

In the present investigation, we have studied for the first time, the aggregation behaviour of a short-chain imidazolium-based IL 1-butyl-3-methylimidazolium octylsulphate [Bmim][OS] within aqueous solutions of deep eutectic solvents ChCl-urea and ChCl-Gly, respectively. We have investigated the role of DESs on the micellization process, *i.e.*, critical micelle concentration (cmc), aggregation number, micellar size and polydispersity index (PDI). A detailed comparative study is performed on the aggregation behavior of IL [Bmim][OS] within the aforementioned two DESs solutions using various spectroscopic techniques. Further, these micellar solutions of [Bmim][OS]-DESs are utilized to study the IL-drug binding of an antidepressant drug promazine hydrochloride (PH).

## 2. Experimental section

### 2.1. Materials

**Scheme 1:**
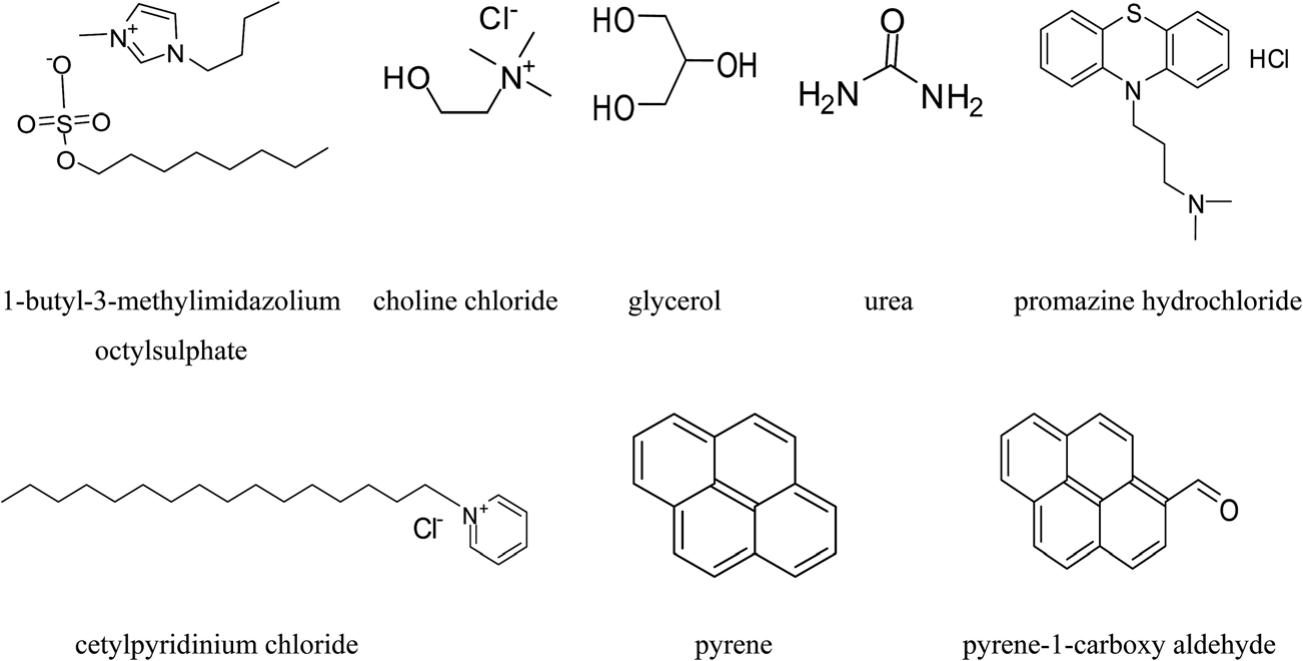
Structures of IL 1-butyl-3-methylimidazolium octylsulphate, choline chloride, glycerol, urea, promazine hydrochloride, cetylpyridinium chloride, pyrene and pyrene-1-carboxyaldehyde.

1-Butyl-3-methylimidazolium octylsulphate IL, potassium bromide, choline chloride, as ammonium salt and urea, glycerol as hydrogen bond donors were purchased from Sigma Aldrich Pvt. Ltd. with high purity and utilized for the synthesis of DESs without further purification. All the aqueous solutions were prepared using millipore water. Chemical structure of IL 1-butyl-3-methylimidazolium octylsulphate, urea, glycerol, pyrene, pyrene-1-carboxyaldehyde, promazine hydrochloride and cetylpyridinium chloride are represented in Scheme 1.

### 2.2. Methods

Fluorescence spectra were performed on ‘’Cary Eclipse spectrophotometer’’ (Agilent Technologies). UV-vis absorption spectra were measured on Cary-60 UV-Vis spectrophotometer (Varian). FT-IR spectra were recorded on Nicolet iS10 spectrometer (Thermo fisher) by using KBr pellets. Dynamic light scattering were performed by Malvern Zeta Sizer Nano (Nano Zs 90 UK).

### 2.3. Preparation and characterization of DESs

**Fig. 1:**
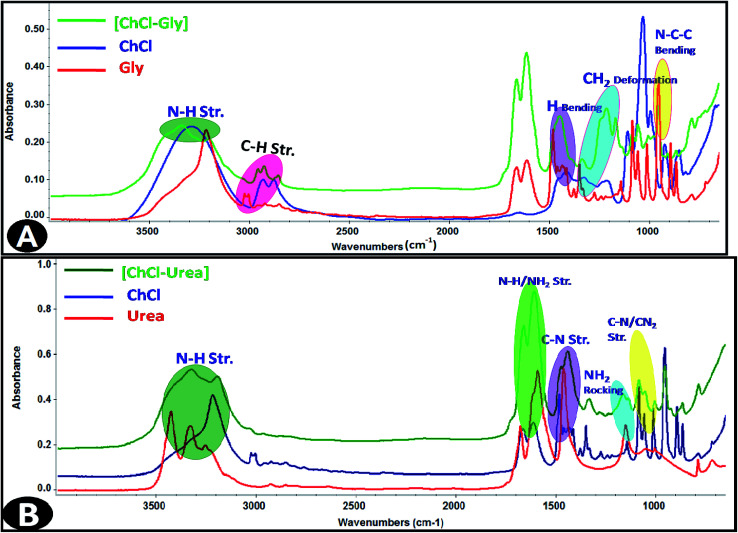
FT-IR spectra of synthesized deep eutectic solvents, (A) ChCl–Gly and (B) ChCl–urea.

In this study, ammonium salt choline chloride (ChCl) and two different hydrogen bond donors (HBDs) namely; urea and glycerol were selected to synthesize the DESs, with different compositions. The deep eutectic solvents were synthesized by mixing the choline chloride salt with different HBDs in 1 : 2 mole fraction of salt at 425 K until a homogenous and colorless liquid appeared. In this study, two types of DESs were synthesized in 1 : 2 ratios of quaternary ammonium salts (choline chloride) with hydrogen bond donors (glycerol and urea, respectively). The eutectic mixtures were prepared by stirring of two components at 425 K until a homogeneous transparent liquid was formed. The structures of the synthetic DESs were confirmed by FT-IR spectral results as shown in the Fig. 1 and data are shown as Table 1.

**Table 1:** A comparisons of the frequencies of the bands observed in IR spectra of glycerol/urea and DESs with their theoretically calculated values

Glycerol				Urea			
Assignments	Observed IR frequencies (cm^−1^)	Calculated frequencies (cm^−1^)	DES frequencies (cm^−1^)	Assignments	Observed IR frequencies (cm^−1^)	Calculated frequencies (cm^−1^)	DES frequencies (cm^−1^)
Asymmetrical NH_2_ stretching	3417	3410	3339	N–H in phase	3317	3328	3328
				Symmetric stretching (O + NH), symmetric stretching CO	1686	1667	1664
C–H stretching	2978	2932	2965	Bending N–H, symmetric bending NH, symmetric bending NH_2_	1629	1592	1611
C–O stretching	2077	2050	2080	Symmetric stretching (C–N), asymmetric stretching CN_2_	1464	1466	1441
N–H bending	1730	1730	1750	Rocking NH_2_	1150	1154	1171
H Bending	1412	1424	1455				
NH bend + CN bend	1341	1345	1335				
CH_2_ deformation	1296	1213	1218	Symmetric stretching (C–N), symmetric stretching CN_2_	1000	1001	1084
C–C stretching + other vibrations	1036	1034	1057	Wagging CO, wagging NH_2_ + CO out of phase	786	789	959
N–C–C bending	936	328	956				

An earlier work of D'Agostino *et al.*^33^ used the pulsed field gradient (PFG) NMR spectroscopic technique to investigate the self-diffusion of molecular and ionic species in aqueous solution of choline chloride (ChCl) based DESs. From the NMR spectrum of aqueous ChCl–Gly at 13 wt% water content, it is shown that the NMR peak positions are (in ppm): *a* = 2.40; *b* = 2.81; *c* = 3.13; *d* = 4.50; e, *f* = 2.67; *g* = 4.17, *h* = 4.25, *i* = 3.68 and for ChCl–urea at 1 wt% water content, the NMR peak positions are (in ppm): *a* = 2.43; *b* = 2.75; *c* = 3.18; *d* = 4.59; *e* = 5.38; *f* = 3.69. It is noteworthy that in aqueous ChCl–urea, the amine species of Ch^+^ and HBD show a stronger interaction with water as water is added to the system. Whereas, in the case of ChCl–Gly, water has little effect on both hydroxyl proton diffusion of Ch^+^ and HBD. Furthermore, Mantle *et al.*^34^ have studied the self diffusion coefficients of the liquid mixtures in a non-invasive way at equilibrium which is measured by pulsed field gradient (PFG)-NMR technique and they have observed that the inter- and intra-dipolar interactions are responsible to origin an enhance effect on the line shapes of the NMR spectrum.

### 2.4. Fluorescence

Steady-state fluorescence experiments are carried out using an Agilent Technology spectrofluorometer. An excitation wavelength of 334 nm is used and emission spectra are scanned between 350 nm to 450 nm. The excitation slit and emission slit width were kept at 2.5 nm. The concentration of probe pyrene (1.2 × 10^−4^ M) and pyrene-1-carboxyaldehyde (1.2 × 10^−4^ M) in the aqueous micellar solutions of [Bmim][OS]–[ChCl–urea]/[ChCl–Gly] are kept fixed.

### 2.5. Dynamic light scattering

The size of amphiphilic micelle was observed by means of dynamic light scattering (DLS) method. DLS measurements were performed with Malvern Zeta Sizer Nano and intensity of the scattered light was maintained at 90° and temperature at 298 K.

### 2.6. Fourier transform infrared spectroscopy

FT-IR spectra of IL 1-butyl-3-methylimidazolium octylsulphate and mixture of choline chloride, urea, glycerol, DESs were obtained using a Nicolet iS10 (Thermo Fisher Scientific Instrument, Nadison, USA) spectrophotometer. All IR spectra were achieved by standard 32 examines at 4 cm^−1^ declaration more the spectral range of 4000–400 cm^−1^. Deep eutectic solvent (DES) mixture with ionic liquid (IL) 1-butyl-3-methylimidazolium octyl sulphate was delivered over 0.1 g pre-weighed finely ground IR grade KBr for DRS-FTIR scan. The KBr was dried around 100 °C, for 5–10 minutes, prior to spectral scan to remove water aberration. The FTIR was purged for 30 minutes with >99.99% analytical grade nitrogen gas using external purge kit (iS10 iZ10 model, Thermo Fisher Scientific), to minimize atmospheric interferences. The dried KBr was then filled over the sample cup and analyte was carefully delivered over it. Diffuse reflectance accessory with IL/DES/KBr beam splitter and deuterated, l-alanine doped triglycinesulphate (DLaTGS) detector was employed in the present work. The software OMNIC 9.1, automatically performs the spectral scaling and the resultant absorptions. The instrument was calibrated as all spectra were obtained by averaging 32 scans at 4 cm^−1^ resolution over the spectral range of 4000–400 cm^−1^ using the auto gain function and slit set at 100 without ATR/DRS modification for wavelength dependence.

### 2.7. UV-visible spectrophotometer

UV-vis absorption spectra were measured using a Varian Cary Eclipsed-60 spectrophotometer. The absorption spectra of the aqueous solution of sort-chain IL + pyrene + DESs mixtures were collected against the reference solutions at 300 K temperature. Pyrene was used as the probe with a concentration of 0.002 (mol dm^−3^) in all experiments.

## 3. Results and discussion

### 3.1. Determinations of critical micelle concentration (CMC)

#### (I) UV-visible spectroscopy

**Fig. 2:**
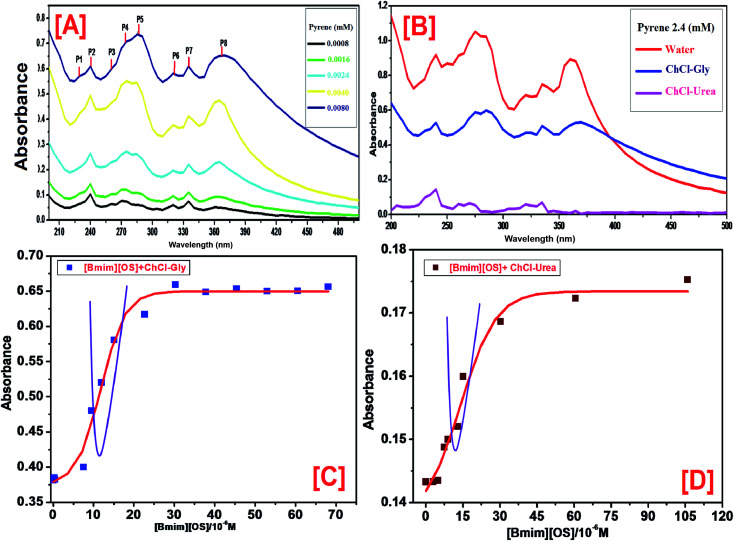
(A) UV-vis spectra of pyrene at different concentration of pyrene, (B) UV-vis spectra of pyrene in 5 wt% ChCl–Gly, ChCl–urea aqueous solution. (C) The plot of absorption intensity *vs.* concentration of [Bmim][OS] in the presence 5wt% ChCl–Gly aqueous solution at the fixed wavelength and (D) the plot of absorption intensity *vs.* concentration of [Bmim][OS] in the presence 5 wt% ChCl–urea aqueous solution at the fixed wavelength (200–600 nm).

The UV-vis absorption spectroscopy is a simple and accurate technique to determine the cmc of various amphiphilic molecules. Pyrene is used as UV probe in our studies. When the concentration of pyrene is changed, the absorption peak heights changed largely as shown in Fig. 2(A). Fig. 2(A) shows that there are total of eight peaks (P1–P8) at 231, 240, 252, 260, 272, 306, 319, and 335 nm in the UV absorption spectra of pyrene in water and the characteristic absorption peaks of pyrene are at 305.5, 319.0 and 334.5 nm. When concentration of IL [Bmim][OS] was increased in the solution, the positions of characteristic peaks shows an obvious red-shift (Fig. 2B) and in the presence of two types of DESs (Fig. 2B). The pre-micellization red-shifts can be attributed to the formation of the IL aggregates just below the cmc. The interaction between these pre-micelles and pyrene result in the red-shifts. Usually, the cmc is determined based on the UV absorption peak, where the centre of the sigmoid is regarded as the cmc. The cmc is defined as the concentration of amphiphilic molecules above which micelles formation takes place and all additional amphiphilic molecules added to the system go to the micelles. After reaching the cmc, there results in a drastic change in the physicochemical properties of the surfactant solution. The cmc is an important characteristic of a amphiphilic molecule. In general, a typical plot of absorbance *versus* concentration of [Bmim][OS] IL is shown in Fig. 2(C) and (D) and the observed cmc of [Bmim][OS] are presented in Table 1. Table 1, clearly shows the cmc of pure IL in water is larger than compared to their values within aqueous mixture of 5 wt% DESs. The results clearly show that the cmc of IL [Bmim][OS] within ChCl–urea DES solution is less than in ChCl–Gly solution.

The formation of the [Bmim][OS] cumulative at the concentration below the cmc, we examined the pre-micellization red shifts. The UV spectra reveal that the red shifts for the strongest UV peak (P2) occur only at the IL concentration 0.1 M. This strong peak is approved to pyrenes that are located at the palisade layer of the micelles and as a result, the red shift of this peak is certified to the close interactions between the IL hydrophilic groups and the π electron clouds of pyrene. As a result, the wavelength *λ*_max_ red shift indicates the formation of micelles. Thus, the ChCl–Gly micelles apply much stronger interactions with the pyrene π electrons than the ChCl–urea micelle.

#### (II) Fluorescence spectroscopy

Fluorescence probes are usually used to achieve various micellar characteristics, such as cmc, aggregation number (*N*_agg_), size and shape, among others. The behavior of a fluorescence probe in a micellar solution depends on the properties of the micellar solution (*e.g.*, nature of the bulk solvent, properties of the micelles, nature of amphiphilic molecules) as well as on the molecular structure of the fluorophore. We have used two fluorescence probes, pyrene and PyCHO to obtain information on DESs added aqueous [Bmim][OS].

##### (A) Behavior of pyrene

**Fig. 3:**
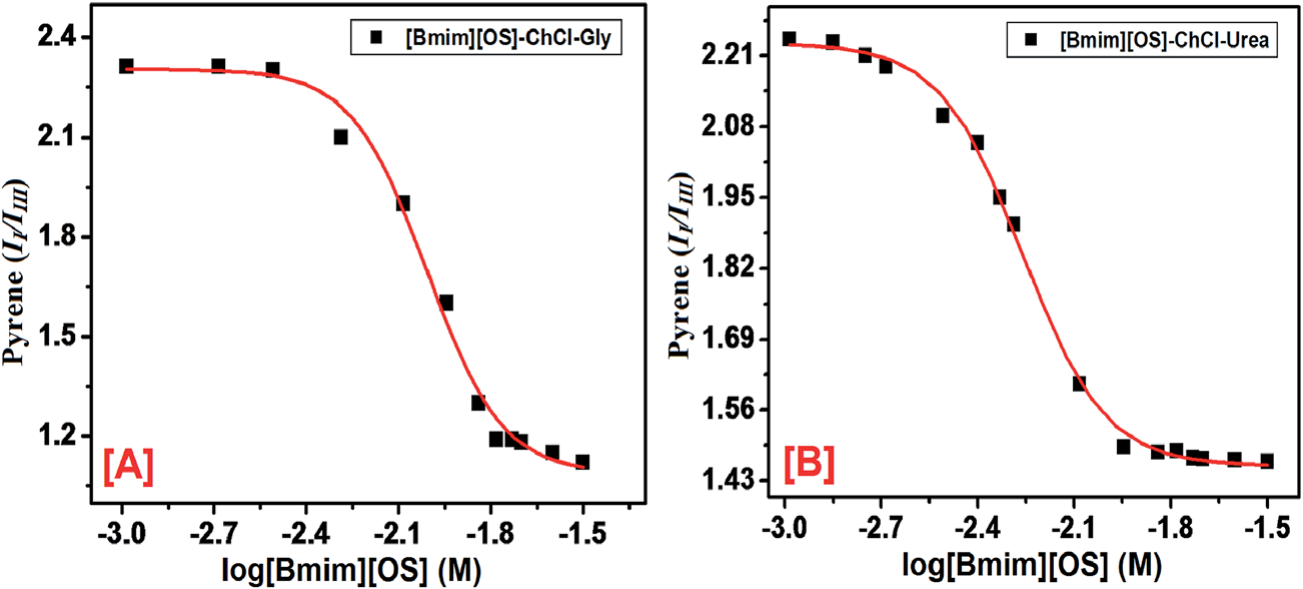
Pyrene (1 μM) *I*_I_*/I*_III_ intensity ratio *vs.* log [Bmim][OS] within 5 wt% aqueous DESs solutions at ambient conditions (*λ*_max_ = 337 nm and slit width 2.5 nm and 1 nm) respectively, [A] [Bmim][OS]–[ChCl–Gly] and [B] [Bmim][OS]–[ChCl–urea].

Fluorescence probe pyrene is a most useful fluorophore to study the self-assembly of IL in DESs. Pyrene has been shown to have two vibronic peaks: (i) first vibronic peak is 373 nm (*I*_I_) and third vibronic peak is 384 nm (*I*_III_) are more sensitive to neighboring medium. The fluorescence spectra of pyrene as a probe within [Bmim][OS] added aqueous solutions of 5 wt% DESs ChCl–urea and ChCl–Gly are shown in Fig. 3. The *I*_I_/*I*_III_ increase very slowly initially since the adsorption of [Bmim][OS] IL molecule at the air–water interface shows the slightly modify in polarity in the bulk. Decreases in *I*_I_/*I*_III_ ratio with the increase in [Bmim][OS] concentration show the movement of pyrene to the hydrophobic non-polar region in the bulk which occurs due to the aggregation of the [Bmim][OS] monomers in the bulk. The area of stability formation once cmc indicates that pyrene resides in the hydrophobic core of the micelle. It is observed that the value of *I*_I_/*I*_III_ is less within the native DESs + water system indicating the residency of pyrene in the hydrophobic region. Also, the cmc obtained for the [Bmim][OS] is higher in water than compared to that in aqueous solution of 5 wt% DESs (ChCl–urea > ChCl–Gly) and the values are in good agreement with those achieved by fluorescence (pyrene as probe) methods. The cmc of [Bmim][OS] in the aqueous solution and in DESs obtained is given in Table 2.

**Table 2:** The critical micelle concentration (cmc) of IL 1-butyl-3-methylimidazolium octyl sulphate in the presence and absence of 5 wt% deep eutectic solvents in aqueous solution by fluorescence and UV-vis spectroscopic methods

DESs	cmc (mM)			Pyrene *I*_I_/*I*_III_
	Fluorescence		UV-vis	
	Pyrene	1-PyCHO	Pyrene	
Water	30.0	28	32	1.00
ChCl–Gly	14.46	14	14	1.12
ChCl–urea	11.36	10	12	1.50

##### (B) Behavior of PyCHO

PyCHO is used as a fluorescence probe to study the micellization behavior of ILs. PyCHO probe shows distinctive structural (Scheme 1) difference that has found usefulness in studies of the solution and interfacial polarity. The fluorescence spectra were recorded keeping fixed concentration of PyCHO (4.1 × 10^−7^ mol L^−1^). PyCHO probe has two types of closely-lying excited singlet states (n–π* and π–π*), both of which show emission in solution. In nonpolar solvents, the emission from PyCHO is highly structured and weak arising from the n–π* state. Change of the dipolarity from non-polar to polar medium the π–π* state is brought below the n–π* state through, solvent relaxation to become the emitting state. This is obvious by a broad reasonably instance emission that red shift with increasing solvent dielectric.

**Fig. 4:**
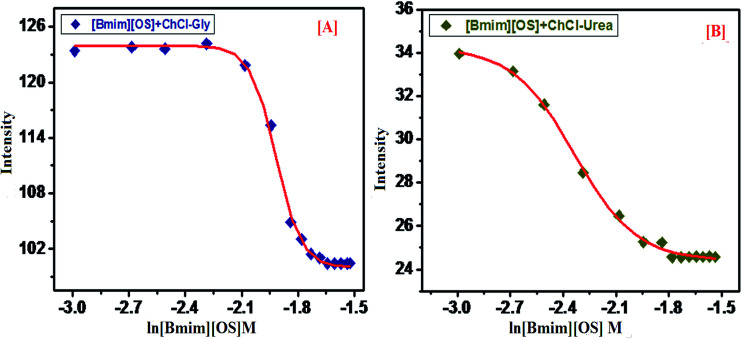
PyCHO (1 μM) intensity *vs.* ln[Bmim][OS] (*M*) concentration in the presence of 5 wt% DESs at ambient conditions (*λ*_max_ = 367 nm and slit width 5 nm) respectively [A] [Bmim][OS]–ChCl–Gly and [B] [Bmim][OS]–ChCl–urea.

PyCHO fluorescence spectra are collected in the presence of varying amount of IL [Bmim][OS] in aqueous 5 wt% DESs solutions. Fig. 4 (S1) shows a hypsochromic shift in *λ*_max_ from pre to the post-micellar region for each addition of signifying, as expected, the increased hydrophobicity of the cybotactic region of the average probe upon micelle formation. Sigmoidal fits to the data are presented using board lines. Leaning among the curves clearly imply early onset of micelle formation in the presence of 5 wt% DESs. It is suggested by our data that PyCHO fluorescence intensity may be more sensitive to the changes in the probe cybotactic region than *λ*_max_. Again as earlier, cmc could be evaluated from the sigmoidal nature of the changes and they are found to be statistically similar to those obtained from pyrene *I*_I_/*I*_III_.

### 3.2. Determination of micellar aggregation number

For the determination of aggregation number (*N*_agg_) of micelles, we have employed steady-state fluorescence quenching method. This has been reported earlier that fluorescence quenching method is better compared to other methods for the determination of *N*_agg_. For a fixed concentration of the [Bmim][OS] IL, the fluorescence intensity decreases with increasing concentration of quencher cetylpyridinium chloride (CPC). The measured ratio of fluorescence intensity in presence of quencher (*F*_Q_) to that in absence of quencher (*F*_0_) is related to the micellar concentration (*M*) by the expression.

The aggregation number of [Bmim][OS] IL micelles in the presence and absence of 5 wt% DESs (ChCl–urea/ChCl–Gly) was obtained through fluorescence quenching of pyrene by a co-surfactant cetylpyridinium chloride (CPC) as quencher according to the Turro–Yekta method:^8^1
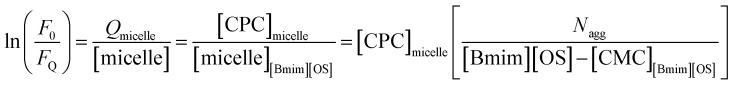
where, *F*_0_ and *F*_Q_ represent the fluorescence intensities of pyrene at 373 nm in the absence and presence of quencher CPC, respectively. [CMC]_micelle_ ([CPC]_micelle_), [micelle]_[Bmim][OS]_ and [Bmim][OS] are the concentrations of quencher (CPC) within the micellar phase, [Bmim][OS] micelles, and [Bmim][OS] IL, respectively. Pyrene (1 μM) fluorescence quenching by CPC in 100 mM aqueous [Bmim][OS] in the absence and presence of 5 wt% DESs according to eqn (1).

**Fig. 5:**
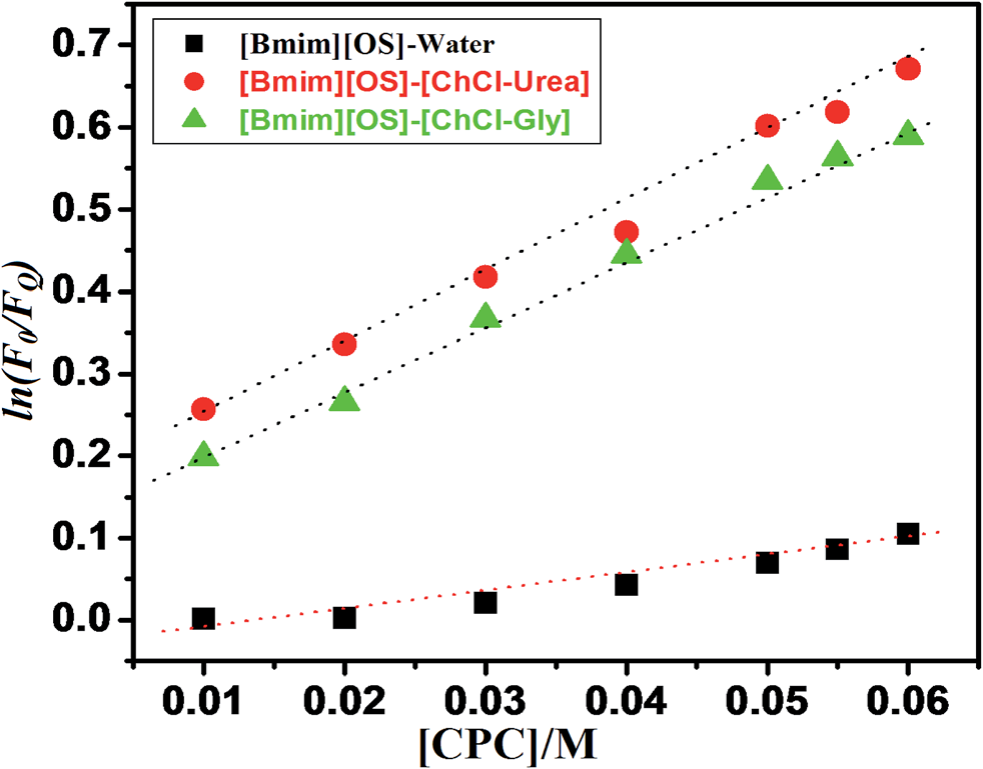
ln(*F*_0_/*F*_Q_) of pyrene (1 μM) as a function of concentration of the fluorescence quenching CPC in 120 mM [Bmim][OS] IL aqueous solution in the presence of 5 wt% DES respectively, 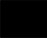 [Bmim][OS]–water, 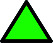 [Bmim][OS]–[ChCl–Gly] and 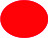 [Bmim][OS]–[ChCl–urea]. Solid lines represent the result of the linear regression analysis.

The aggregation number can be calculated from the slope of ln(*F*_0_/*F*_Q_) *vs.* [CPC]_micelle_ plots at a fixed CMC_IL_, Fig. 5, displays the ln(*F*_0_/*F*_Q_) as a function of [CPC] in [Bmim][OS] aqueous solutions and the good linear correlation are obtained. The relevant *N*_agg_ value was obtained by applying eqn (1) and these values are listed in Table 3. It can be seen that the *N*_agg_ of [Bmim][OS] decreased on going to ChCl–Gly and ChCl–urea DESs.

**Table 3:** The aggregation number (*N*_agg_), Stern–Volmer constants (*K*_sv_), hydrodynamic radii (Rh) and polydispersity index (PDI) of IL 1-butyl-3-methylimidazolium octylsulphate in the presence and absence of 5 wt% deep eutectic solvents in aqueous solution by fluorescence method

DES	*N*_agg_	*K*_sv_	Rh (*d* nm)	PDI
Water	19	0.85	220, 40	0.56
ChCl–Gly	134	8.13	135, 20	0.97
ChCl–urea	163	8.29	209, 48	0.72

Arvind *et al.*^23^ also studied the effect of derivative on ethylene glycol with [Bmim][OS], addition of various wt% of EG/EGMME/EGDME as reported in ref. 23. The reported, *N*_agg_ of 22 (10 wt% EG), 20 (30 wt% EG), 20 (10 wt% EGMME), 16 (30 wt% EGMME), 18 (10 wt% EGDME) and 17 (30 wt% EGDME). The *N*_agg_ of [Bmim][OS] within aqueous DESs is much higher than that of [Bmim][OS] in water. It may be attributed to the different proton donor and some proton acceptor based DESs. It was found that *N*_agg_ were (19, 134 and 163) for pure [Bmim][OS] in water and 5 wt% aqueous DESs [Bmim][OS] + ChCl–Gly, [Bmim][OS] + ChCl–urea solution, respectively. This may be caused by: (1) the larger aggregate size in aqueous solutions, this result being accordant with the trend of β and also (2) in present work, we had found the ln(*F*_0_/*F*_Q_) value of [Bmim][OS] was lesser than that of mixture of DESs with [Bmim][OS] (water < ChCl–Gly < ChCl–urea). This may be due to the close packing of [Bmim][OS] molecular in their micelle, which causes the water molecules to penetrate into the micelle and may lead to the bending of the long rigid hydrophobic part.

The strength of the hydrophobic environment of short-chain based IL can be estimated by the Stern–Volmer quenching constant (*K*_sv_) was calculated using the following eqn (2);

2 ln *F*_0_/*F*_Q_ = 1 + *K*_sv_[Q]

The Stern–Volmer quenching constant (*K*_sv_) can be probable from the reached slope values of the plots ln *F*_0_/*F*_Q_*versus* [CPC]. The calculated *K*_sv_ values are illustrated in Table 3. *K*_sv_ are explained the hydrophobicity of micellar solutions and its utilized to decrease the fluorophore. Table 3, are clearly show the *K*_sv_ value of pure IL are lesser compared to mixed of IL-DESs (ChCl–urea > ChCl–Gly). The result shows that the broad behavior of the micellar aggregation of [Bmim][OS] in ChCl–urea is comparable to that in water, *i.e.* the micellization of [Bmim][OS] in ChCl–urea is mostly determined by the solvophobic effect, similar to the micellization of IL molecules in water caused by the hydrophobic effect.^32^

### 3.3. Particle size from dynamic light scattering

**Fig. 6:**
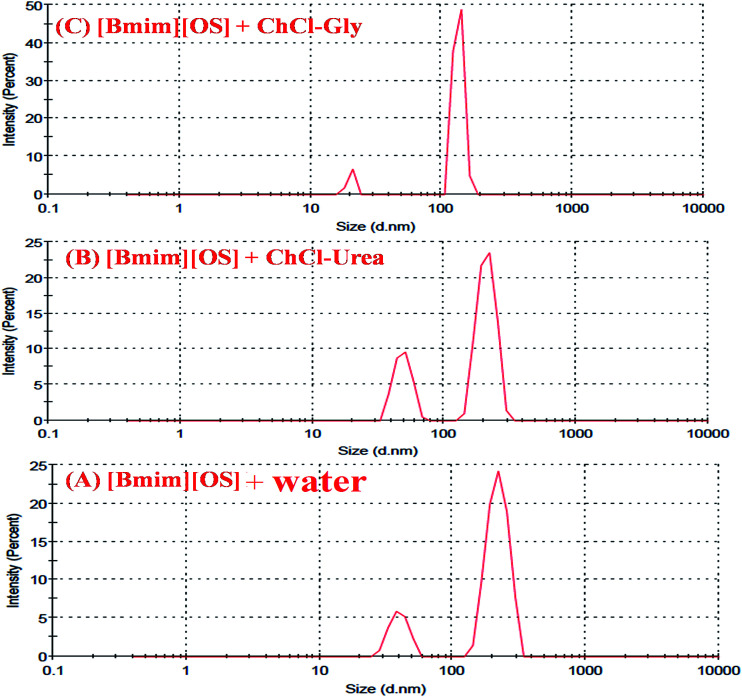
Aggregate size distribution obtained from DLS at 298 K and in the presence of 5 wt% deep eutectic solvents in aqueous [Bmim][OS] *i.e.*, (A) [Bmim][OS]–water, (B) [Bmim][OS]–ChCl–urea and (C) [Bmim][OS]–ChCl–Gly.

Dynamic light scattering (DLS) technique was employed to investigate the micro structural changes taken place within the IL-added DESs-based systems. Successive measurements were made within a cell of 3 mL for normalization analysis. The average hydrodynamic radius distribution of the DESs solution (0.1 g mL^−1^), [Bmim][OS] solution (0.2 mg mL^−1^) and DES-rich phase after extraction (diluted 5 times) are shown in Fig. 6. DLS was useful technique to substantiate the evidence of aggregate formation in aqueous imidazolium based IL [Bmim][OS] in presence and absence of 5 wt% aqueous DESs and also to study the variation of size of aggregates in the system. Fig. 6 shows the scattering intensity for the given diameter (*D*), PDI measured at room temperature of aqueous [Bmim][OS] and in the presence of DESs.

The bimodal distribution and PDI is observed and shown in Table 3. Our DLS results are in the evidence of the formation of micelle like aggregates of [Bmim][OS] even in the presence of DESs. The average radius of pure amphiphilic IL is hydrodynamic radii larger Rh = 220, 40 (*d* nm) and a polydispersity index PDI = 0.56. The complexation of IL with DESs of (a) ChCl–urea is Rh = 209, 48 (*d* nm), PDI = 0.72, (b) ChCl–gly is Rh = 135, 20 (*d* nm), PDI = 0.97 respectively. This observation suggests that DES is bound to [Bmim][OS] and the DES–[Bmim][OS] complex was formed. The combination was due to the electrostatic interaction of ChCl–urea with [Bmim][OS] and the strong hydrophobic interactions between the hydrocarbon chains of ChCl–Gly and hydrophobic bases of [Bmim][OS]. The overall studies show the evidence of formation of micelle like aggregates even in the presence of DES.

### 3.4. Fourier transform infrared (FTIR) spectroscopy

**Fig. 7:**
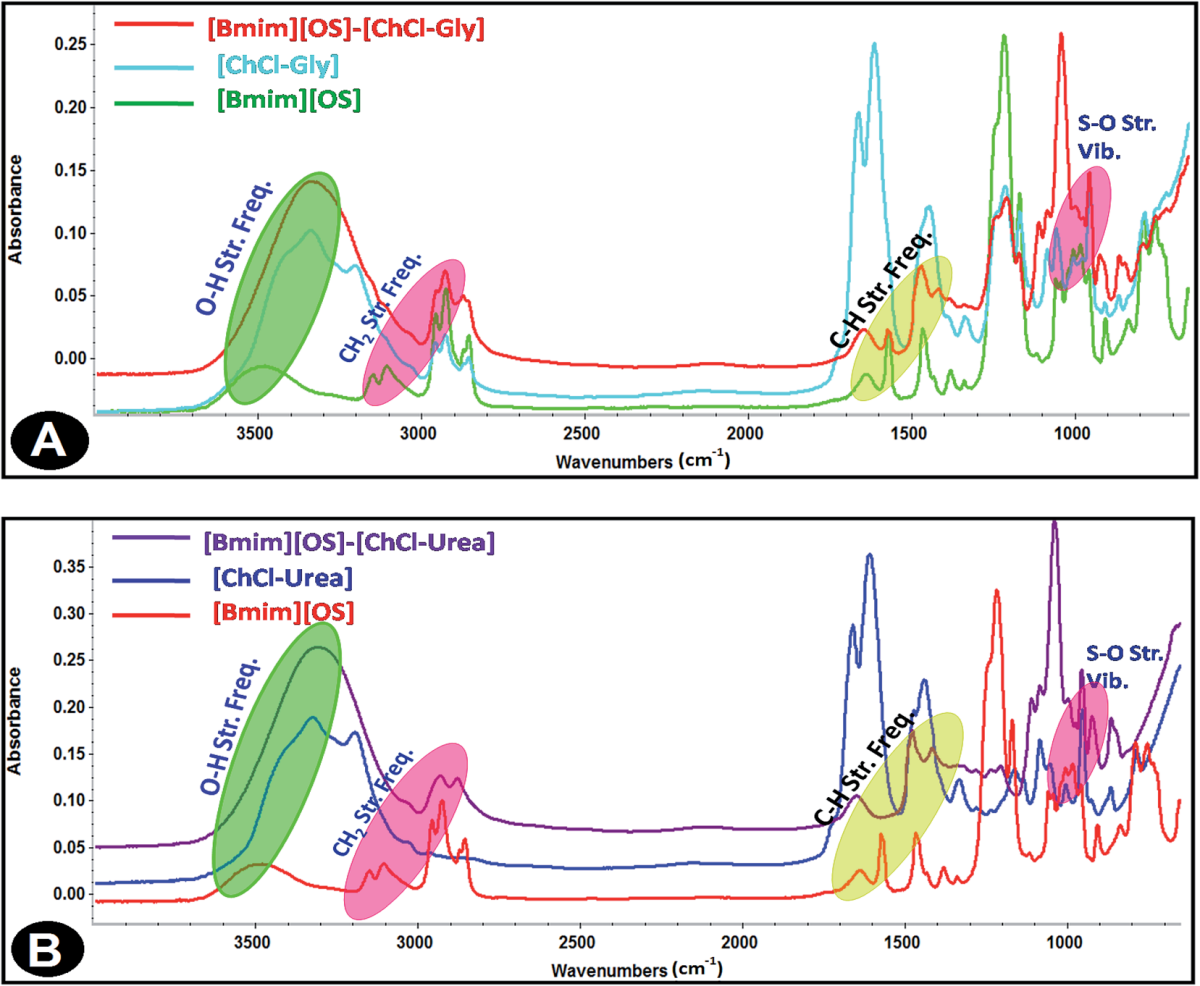
FT-IR spectra of 5 wt% DESs aqueous solutions (A) [Bmim][OS]–ChCl–Gly and (B) [Bmim][OS]–ChCl–urea.

Infrared spectroscopy is a standard analytical tool for assessing liquid structures. The intra-molecular vibrational modes of the ions composing the materials are often quite sensitive to their local potential energy environment. FT-IR spectroscopy is a characteristic technique to analyze the strength of Hydrogen bond interactions and identify the structure of DESs between hydrogen bond donor (HBD) and hydrogen bond acceptor (HBA). In the present study, FT-IR spectral response is used to achieve the hydrogen bond interactions taking place within IL-added DES solutions based on the spectral shifts. The FT-IR spectra of the [Bmim][OS]/ChCl–Gly/ChCl–urea micellar solutions are shown in Fig. 7.

The FT-IR spectra of 1-butyl-3-methylimidazolium octylsulphate (symmetric and asymmetric stretching CH_2_ vibration of alkyl chains) at 2856.19 cm^−1^, 2926.19 cm^−1^ is shifted to 2876.69 cm^−1^, 2929.69 cm^−1^, (symmetric and asymmetric stretching C–H scissoring vibration of CH_3_–moiety) at 1466.13 cm^−1^ is shifted to 1472.30 cm^−1^, (symmetric S–O stretching vibration) 982.77 cm^−1^ shifted to 959.59 cm^−1^ in [Bmim][OS]–ChCl–Gly complex. The IR spectra of 1-butyl-3-methylimidazolium octylsulphate (Fig. 7) (symmetric and asymmetric stretching CH_2_ vibration of alkyl chains) at 2856.19 cm^−1^, 2926.19 cm^−1^ are shifted to 2885.05 cm^−1^, 2937.99 cm^−1^, (symmetric and asymmetric stretching C–H scissoring vibration of CH_3_–moiety) and 1466.13 cm^−1^ is shifted to 1483.45 cm^−1^, (symmetric S–O stretching vibration) 982.77 cm^−1^ shifted to 954.09 cm^−1^ in [Bmim][OS]–ChCl–urea complex. The development of the hydrogen bonding strength depicts [Bmim][OS] molecules distributed in the aggregates closely mutually and supports the dissociation of head group counter ions in the surface of aggregates, resulting in a closer arrangement of micelles.

### 3.5. Ionic liquid-drug binding of promazine hydrochloride

**Fig. 8:**
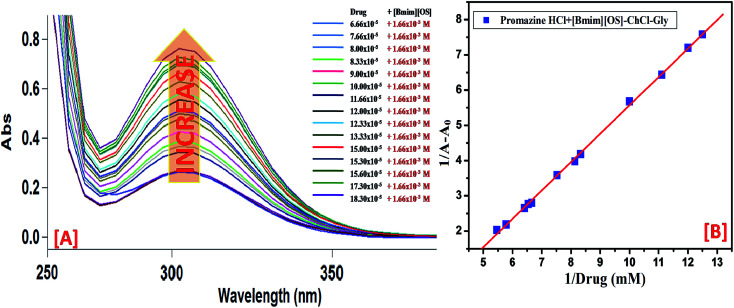
Absorption spectra of PH with increasing concentration of (A) [Bmim][OS] and (B) Benesi–Hildebrand plot using changes in absorption spectra of Drugs–[Bmim][OS]–ChCl–Gly.

UV-visible absorption spectroscopic technique is a constructive tool to probe IL-drug binding.^35^ In the absorption spectra of drug, absorbance at 300 nm increases upon addition of micellar solution ([Bmim][OS] (100 mM)-ChCl–Gly/ChCl–urea) (Fig. 8). The peak positioned around 300 nm is red shifted. Based on the peak shift and increase in absorbance, it can be concluded that all mixture can form complex with the PH drug since ChCl–Gly and ChCl–urea have almost no absorption band through the wavelength range (300–600 nm) (Fig. 8). The absorption band for the promazine hydrochloride (10 mM) drug was observed at *λ*_max_ = 300 nm.

**Scheme 2:**
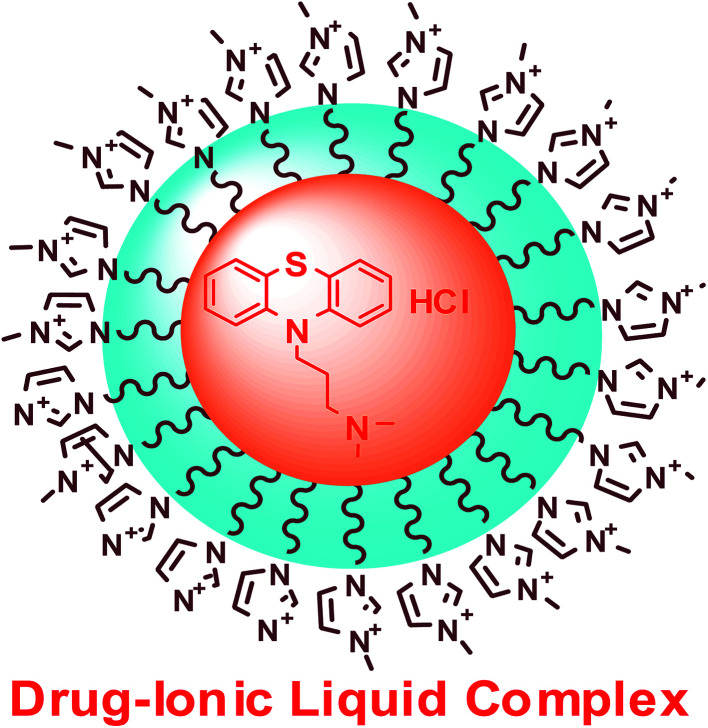
Interaction of 1-butyl-3-methylimidazolium octylsulphate within deep eutectic solvent.

The binding constants for drug–IL complexes were estimated from Benesi–Hildebrand (B–H) equation. The change in absorbance is depended on the concentration of drug, according to the following eqn (3),3
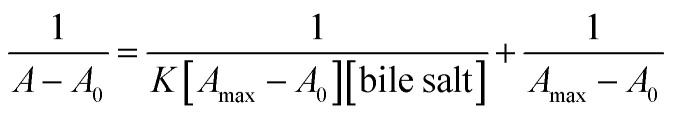
where, *A*_0_, *A* and *A*_max_ are the absorbance in the absence of IL, at intermediate concentration of IL, at saturation point, respectively and *K* is the binding constant. The plot of 1/[*A* − *A*_0_] *vs.* 1/[drug] gives straight lines (Fig. 8), which further indicates the formation of 1 : 1 complex between drug (PH) and IL representation on Scheme 2. The values of the binding constant obtained from the intercept-to-slope ratio of the Bensei–Hildebrand plot (Fig. 8 and S2†) for drug/IL complexes show that the ChCl–urea (6 mol dm^−1^) shows more binding affinity towards PH than ChCl–Gly (5 mol dm^−1^) as they readily interact and form hydrogen bond with water. Owing to substituted hydroxyl group, it reduces the unfavorable interactions.

## 4. Conclusions

We have investigated the micellization behavior of a short-chain IL [Bmim][OS] within two DESs ChCl–urea and ChCl–Gly using UV-vis, fluorescence, DLS and FT-IR spectroscopic techniques. Significant decrease in cmc values and increase in *N*_agg_ in aqueous DESs solutions indicate an overall constructive micellization process. This is explained on the basis of enhanced hydrophobic and electrostatic interactions within [Bmim][OS]–DESs systems, a behavior similar to that of electrolytes. The cmc value of IL [Bmim][OS] in aqueous solution of DES ChCl–Gly is observed to be larger than that in ChCl–urea solution, which shows the importance of HBD in favoring the micellization of IL in the solution. DLS results indicate that while the size distribution of the [Bmim][OS] micelles in aqueous solution is narrow, the assemblies of IL formed in aqueous ChCl–urea compared to ChCl–Gly are relatively widely distributed. The strength of hydrogen bond interaction between ChCl and HBD (glycerol and urea) were investigated from FT-IR spectral responses. The solvophobic effect dominates the micellization of [Bmim][OS] in aqueous DESs and the intermolecular hydrogen-bond interaction plays a positive role to promote the micellization process. PH drug shows more binding affinity and most capable action is shown by ChCl–urea over ChCl–Gly. The present work clearly shows the tendency to form self assembled nanostructures by short-chain imidazolium IL within aqueous DESs and this would serve for potential application of IL- and DES-based systems in drug delivery, aggregation, colloidal systems and novel ways for researchers to explore new findings.

## Conflicts of interest

There are no conflicts to declare.

## Supplementary Material

RA-008-C7RA13557B-s001
