# Supplementary material for: Self-assembly of a short-chain ionic liquid within deep eutectic solvents
Source: RSC Adv. 2018 Feb 20;8(15):7969–79. doi: 10.1039/c7ra13557b (PMC9078456; doi:10.1039/c7ra13557b)
Supplement: RA-008-C7RA13557B-s001 [file RA-008-C7RA13557B-s001.pdf]

Electronic Supplementary Material (ESI) for RSC Advances.
This journal is © The Royal Society of Chemistry 2018

Supporting Information (S)

Fig. S1 PyCHO (1μM) intensity versus log [Bmim][OS] (M) at ambient conditions (λ_{max} = 367 nm and slit width 5nm)

Fig. S2 Absorption spectra of PH with increasing concentration of (A) Drugs-[Bmim][OS]-ChCl-Urea and (B) Benesi–Hildebrand plot using changes in absorption spectra of Drugs-[Bmim][OS]-ChCl-Urea.

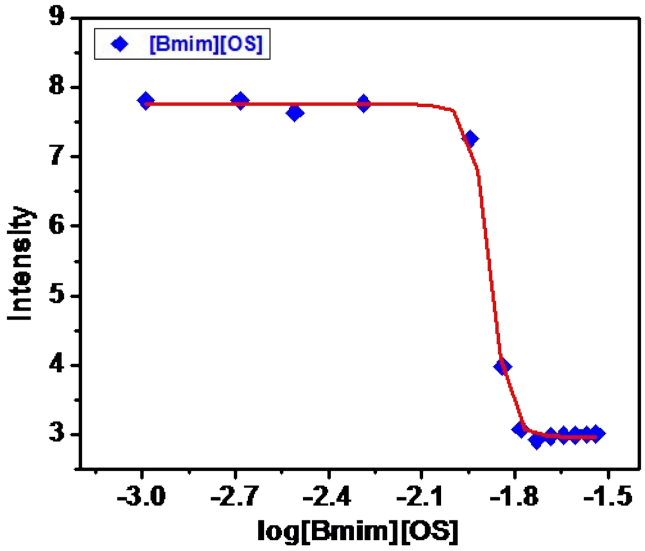

Fig. S1 PyCHO (1μM) intensity versus log [Bmim][OS] (M) at ambient conditions (λ_{max} = 367 nm and slit width 5nm)

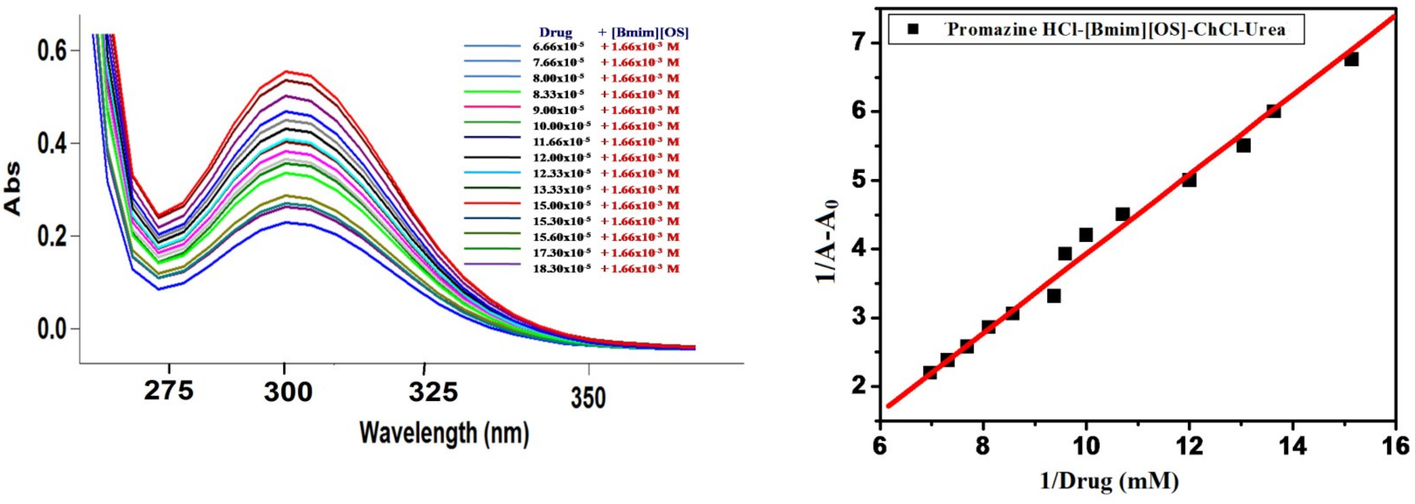

[A] **[B]**

Fig. S2 Absorption spectra of PH with increasing concentration of (A) Drugs-[Bmim][OS]-ChCl-Urea and (B) Benesi–Hildebrand plot using changes in absorption spectra of Drugs-[Bmim][OS]-ChCl-Urea.
